# The addition of a goal-based motivational interview to standardised treatment as usual to reduce dropouts in a service for patients with personality disorder: a feasibility study

**DOI:** 10.1186/1745-6215-11-98

**Published:** 2010-10-14

**Authors:** Mary McMurran, W Miles Cox, Stephen Coupe, Diane Whitham, Lucy Hedges

**Affiliations:** 1 Institute of Mental Health, University of Nottingham, Sir Colin Campbell Building, Triumph Road, Nottingham NG7 2TU, UK; 2 School of Psychology, Bangor University, Adeilad Brigantia, Bangor, Gwynedd LL57 2AS, UK; 3 Personality Disorder Network, Nottinghamshire Healthcare NHS Trust, Mandala Centre, Gregory Boulevard, Nottingham NG7 6LB, UK; 4 Clinical Trials Unit, University of Nottingham, Office B39, Medical School, Queens Medical Centre, Nottingham NG7 2UH, UK

## Abstract

**Background:**

Rates of non-completion of treatments for personality disorder are high and there are indications that those who do not complete treatment have worse outcomes than those who do. Improving both cost-efficiency and client welfare require attention to engaging people with personality disorder in treatment. A motivational interview, based on the Personal Concerns Inventory, may have the ability to enhance engagement and retention in therapy. Here, we report the protocol for a feasibility study for a randomised controlled trial (RCT).

**Methods:**

All referrals accepted to the psychological service of Nottinghamshire Healthcare NHS Trust's outpatient service for people with personality disorder are eligible for inclusion. Consenting participants are randomised to receive the Personal Concerns Inventory interview plus treatment as usual or treatment as usual only. We aim to recruit 100 participants over 11/2 years. A randomised controlled trial will be considered feasible if [1] the recruitment rate to the project is 54% of all referrals (95% CI 54-64), [2] 80% of clients find the intervention acceptable in terms of its practicability and usefulness (95% CI 80-91), and [3] 80% of therapists report finding the intervention helpful (95% CI 80-100). In a full-scale randomised controlled trial, the primary outcome measure will be completion of treatment i.e., entry into and completion of ≥ 75% of sessions offered. Therefore, information will be collected on recruitment rates, attendance at therapy sessions, and completion of treatment. The feasibility of examining the processes of engagement will be tested by assessing the value, coherence, and attainability of goals pre-treatment, and engagement in treatment. The costs associated with the intervention will be calculated, and the feasibility of calculating the cost-benefits of the intervention will be tested. The views of clients and therapists on the intervention, collected using semi-structured interviews, will be analysed using thematic analysis.

**Discussion:**

The Personal Concerns Interview as a preparation for treatment of people with personality has the potential to maximise treatment uptake, reduce unfilled places in treatment programmes, and prevent group treatments faltering through non-attendance. Most importantly, it has the potential to improve patient outcomes, helping them to function better and reduce hospitalisation.

**Trial Registration:**

ClinicalTrials.Gov.UK Identifier - NCT01132976

## Background

In the UK in recent years, there has been recognition that people with personality disorders have been poorly served by mental health services, and so, in 2003, the Department of Health issued a directive to develop services for people with personality disorders in a document entitled, *Personality Disorder: No Longer a Diagnosis of Exclusion *[1]. In 2009, the National Institute for Clinical Excellence (NICE) published guidelines for the treatment and management of borderline personality disorder and antisocial personality disorder (see http://www.nice.org.uk). Clearly, there are significant developments in provision for people with personality disorders. One area that needs to be considered when developing treatments and services is that of promoting client attendance and engagement.

In a recent systematic review of non-completion of treatments for personality disorder, McMurran, Huband and Overton [2] identified that, on average, 37% do not complete their treatment. This level of non-completion compromises service efficiency and cost-effectiveness. More importantly, there is evidence that non-completion of treatment is associated with poorer outcomes. Compared with treatment completers, people diagnosed with personality disorder who do not complete treatment have been shown to be hospitalised more frequently and spend more days in hospital [3,4]. Efforts need to be made to engage people with personality disorder in therapy.

In a recent systematic review of strategies for reducing drop-out rates in psychotherapy generally, only 15 empirical studies were identified [5]. Of these, 12 were pre-therapy preparation (role induction, experiential pre-training), and half of these studies had positive outcomes on retention in treatment. Two types of intervention mentioned as potentially effective but not evaluated were negotiating the goals of therapy and motivational enhancement. Furthermore, the author of the review commented on a need to identify strategies that are effective with specific groups of patients and mentioned that "patients with severe personality disorder are notoriously difficult to keep engaged in treatment..... Identifying effective strategies for keeping these patients in therapy would have a major clinical impact" ([5], p. 68).

One promising approach that assists the therapist to motivate people to engage in therapy and effect positive change is based upon identification of valued goals. In evolutionary terms, goals are specific representations of what is needed for survival, and goal pursuit refers to the range of activities employed in the quest for goal attainment [6]. Needs range from basic physiological needs (e.g., oxygen, food, water), through needs for safety and belonging, to higher order needs for esteem and self-actualisation. Personal goals are what give purpose, structure and meaning to a person's life [7], and well-being is experienced when there is commitment to goal attainment, goals are achievable, and goals meet the individual's explicit and implicit needs [8].

One specific theory of motivation in which goal striving plays a central role is the Theory of Current Concerns [9]. Within this framework, each goal pursuit corresponds to an internal state called a 'current concern'. Goals are identified and rated on scales of value, attainability, control and commitment in an interview called the Personal Concerns Inventory [10]. The rating scales provide information that enables the calculation of indices representing a person's motivational structure. Empirical investigations have revealed adaptive and maladaptive motivational profiles [11,12]. The adaptive motivation factor is characterised by high perceived likelihood of goal attainment, expected happiness when goals are attained, and commitment to goal striving, and is predictive of readiness to change and reduction of problem behaviours.

Although the Personal Concerns Inventory is an assessment of goals and motivational structure, the experience of clarifying one's goals and values can be beneficial in itself, and there is some evidence that engaging in the interview may motivate people to enter treatment [13]. This effect might be capitalised upon by developing the Personal Concerns Inventory procedure into a more fully rounded motivational interview and evaluating its effect. The research proposed here is the start of this process and the target population is people in treatment for personality disorder with Nottinghamshire Healthcare NHS Trust's outpatient service for people with personality disorders. In this service, 354 people were offered psychological treatment between 2005 and 2008. Of these, 31% dropped out of treatment prematurely.

### Study aims

The primary aim of this research is to gather information that will determine whether a randomised controlled trial (RCT) to evaluate the effectiveness of a goal-based motivational intervention called the Personal Concerns Inventory in a community personality disorder treatment service is feasible. Specifically, we aim to [1] measure the recruitment rate to the Personal Concerns Inventory interview plus treatment as usual or treatment as usual only, and [2] assess the acceptability of the intervention to clients and therapists.

## Methods and Design

### Design

This is a feasibility study for a randomised controlled trial (RCT).

### Ethics

Approval for the research was given by the Leicestershire, Northamptonshire & Rutland Research Ethics Committee 1 (Ref: 09/H0406/76) and Nottinghamshire Healthcare NHS Trust's Research Management and Governance Section (Ref: CSP/18/05/10 CSP ID 19434).

### Participants and Recruitment

Nottinghamshire Healthcare NHS Trust offers an outpatient service to people with personality disorder. This service has three tiers: 1. information and advice, 2. a 16-week psychological intervention based upon psychoeducation, social problem solving and emotion regulation; and 3. a long-term therapeutic community. All referrals accepted to the psychological intervention are eligible for inclusion. Those who are accepted for treatment are informed about the project and asked if they are willing to meet with the researcher to receive further information. Potential participants are fully informed about the research and given an information sheet. Consent to participate is taken by the individual's clinician at the next appointment. After assessment and psychoeducation, which is the first part of usual treatment, participants are randomised to receive the Personal Concerns Inventory interview plus treatment as usual or treatment as usual only. The Personal Concerns Inventory is completed by the service's therapists, all of whom have been trained in its delivery. The number of people assessed for psychological treatment by the service is 118 per year; we aim to recruit 100 participants over 11/2 years.

### Randomisation and Blinding

Randomisation is based on a computer-generated pseudo-random code using random permuted blocks of randomly varying size, created by the Nottingham Clinical Trials Unit (CTU) in accordance with their standard operating procedure and held on a secure server. Treatment allocation for each participant is accessed by means of a remote, internet-based randomisation system developed and maintained by the Nottingham CTU. The sequence of treatment allocations are concealed from the Research Assistant responsible for administering the outcome measures until trial-related assessments are complete.

### Interventions

**Figure 1:**
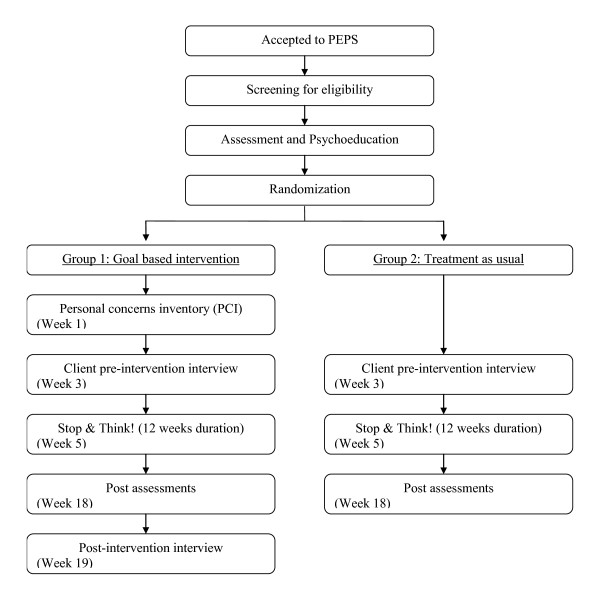
**The intervention and assessment schedule flowchart**.

The comparison is between a goal-based motivational interview called the Personal Concerns Inventory plus treatment as usual and and treatment as usual only. Treatment as usual is up to four individual weekly sessions of psychoeducation, based on personality assessment and information exchange [14], after which there is a weekly problem solving therapy group lasting 12 weeks [15]. The intervention and assessment schedule is presented in Figure 1.

Participants recruited to the Personal Concerns Inventory group receive one or two interviews of maximum total duration of 3 hours after assessment and receipt of the psychoeducation component of treatment. The Personal Concerns Inventory procedure asks participants to identify their goals in 11 life areas (e.g., relationships, work or education, home, health), and then prioritise five goals. These five goals are then rated on scales from 0 to 10 assessing five aspects of goal attainment: likelihood of attainment, control over attainment, knowing how to attain it, happiness upon attainment, and commitment to attaining it. Participants are asked to identify obstacles to goal attainment and consider the possibility that therapy could help them overcome these obstacles. This is intended to enhance participants' motivation to engage in therapy.

### Assessments

Part of the service's routine assessment is administration of the Personality Diagnostic Questionnaire-4 (PDQ-4;16), a 100-item, self-administered, true/false questionnaire that yields personality diagnoses consistent with the DSM-IV diagnostic criteria for the axis II disorders. Information from this will be used to describe the personality disorder profile of the sample.

Within the two weeks prior to the start of the problem solving therapy group, participants in both the Personal Concerns Inventory group and the treatment as usual group are interviewed briefly to assess the goals that they expect therapy to help them achieve. The goals generated by participants will be rated for quality (i.e., clarity, attainability, value). This information is designed to tell us whether the Personal Concerns Inventory works better than treatment as usual to clarify clients' thinking about their goals and the potential value of therapy in assisting with goal attainments. Throughout the problem solving group therapy, attendance records are kept and at the end of group therapy, therapists rate each participant's level of engagement using the Treatment Engagement Rating Scale (TER;17). This scale contains items addressing the client's participation, constructive use of sessions, opennness, efforts to change, making sacrifices, goal directedness, and reflection.

Information is collected about recent receipt of services throughout using the Client Service Receipt Inventory (CSRI; 18). The CSRI captures recent use of health and social care. The CSRI is administered at baseline and again at the end of treatment.

At the end of treatment, participants in the Personal Concerns Inventory group, both treatment completers and non-completers, are interviewed, either by a researcher or by a service user, to ask for their views on the acceptability and usefulness of the interview. Therapists are also interviewed to assess their opinions of the Personal Concerns Inventory.

### Analyses

A randomised controlled trial will be considered feasible if [1] the recruitment rate to the project is 54% of all referrals (95% CI 54-64), [2] 80% of clients find the intervention acceptable in terms of its practicability and usefulness (95% CI 80-91), and [3] 80% therapists report finding the intervention helpful (95% CI 80-100). In a full-scale randomised controlled trial, the primary outcome measure will be completion of treatment i.e., entry into and completion of ≥ 75% of sessions offered. Therefore, information will be collected on recruitment rates, attendance at therapy sessions, and completion of treatment. The feasibility of examining the processes of engagement will be tested by assessing the value, coherence, and attainability of goals pre-treatment, and engagement in treatment (TER). The costs associated with the intervention will be calculated, and the feasibility of calculating the cost-benefits of the intervention will be tested (CSRI). The views of clients and therapists on the intervention, collected using semi-structured interviews, will be analysed using thematic analysis [19].

## Discussion

The means whereby the Personal Concerns Inventory should work to improve engagement and retention in therapy is by helping the interviewee identify what are his or her important goals, the obstacles to goal attainment, and the relevance of psychological therapy to overcoming obstacles. The end result of therapy should, therefore, be a greater likelihood of attaining important goals and improving life satisfaction.

If the Personal Concerns Interview administered in preparation for treatment of people with personality disorder improves engagement and retention in treatment, then this could be an economical way of improving the cost-effectiveness of treatments. It has the potential to maximise treatment uptake, reduce unfilled places in treatment programmes, and prevent group treatments faltering through non-attendance. Most importantly, it has the potential to improve patient outcomes, helping them to function better and reduce hospitalisation.

## Competing interests

The authors declare that they have no competing interests.

## Authors' contributions

MM, WMC, SC, and DW contributed to the design of the study. All authors contributed to the creation of the Manual of Procedures and the study protocol. MM drafted the manuscript. All authors provided a critical review and final approval of the manuscript.
